# Case Report: Sequential leadless pacing after battery depletion in a patient with recurrent device-related infection

**DOI:** 10.3389/fcvm.2026.1856082

**Published:** 2026-07-21

**Authors:** Assel Chinybayeva, Gaziza Marat, Zhanasyl Suleymen, Ayan Abdrakhmanov

**Affiliations:** 1 Heart Rhythm Scientific Research Institute, NpJSC “Astana Medical University”, Astana, Kazakhstan; 2 Heart Rhythm Center, National Coordination Center for Emergency Medicine, Ministry of Healthcare of the Republic of Kazakhstan, Astana, Kazakhstan

**Keywords:** battery depletion, cardiac implantable electronic device infection, case report, leadless pacemaker, sequential pacing

## Abstract

**Background:**

Leadless pacemakers offer an important alternative for patients with recurrent cardiac implantable electronic device infections. However, clinical data on long-term management and sequential implantation strategies after battery depletion remain limited. We aimed to evaluate the feasibility and safety of a sequential leadless-only pacing strategy in a patient with a multi-decade history of pacing-related complications.

**Case Presentation:**

We report the long-term clinical course of a patient with a 26-year history of cardiac pacing (2000–2026). Following multiple transvenous system failures and recurrent infections, the patient transitioned to a leadless platform in 2017. We describe the clinical rationale and procedural outcome of upgrading from a depleted first-generation leadless pacemaker (Micra VR, implanted 2017, left *in situ* at the right ventricular septum) to an AV-synchronous leadless pacemaker (Micra AV, implanted 2025 because of battery depletion of the prior device, not infection) using a leave-in-situ approach.

**Results:**

Sequential implantation was successfully performed in 2025, with the new device implanted at the right ventricular mid-septum while the previously implanted device was left *in situ*. Acute electrical parameters were satisfactory (pacing threshold 0.63 V, R-wave amplitude 9.8 mV, impedance 720 *Ω*), with no evidence of inappropriate sensing. At follow-up, electrical parameters remained stable (pacing threshold 0.50 V, impedance 670–650 *Ω*), with atrioventricular synchrony of 80% at 1 month and 84–85% at 3–6 months. No clinical or device-telemetry evidence of mechanical interaction or electrical interference between the devices was observed during follow-up. No device-related complications occurred during follow-up.

**Conclusion:**

A sequential leadless-only pacing strategy, including a leave-in-situ approach, may represent a feasible and safe long-term option in selected high-risk patients with device-related infections and a history of transvenous system extraction.

## 1 Introduction

Leadless pacing systems have become an established option for patients at high risk of device-related infection (1, 2). By eliminating the subcutaneous pocket and transvenous leads, leadless pacing systems reduce the risk of cardiac implantable electronic device infection (3, 4), although isolated cases of leadless pacemaker infection have nonetheless been reported (5).

**Figure 1:**
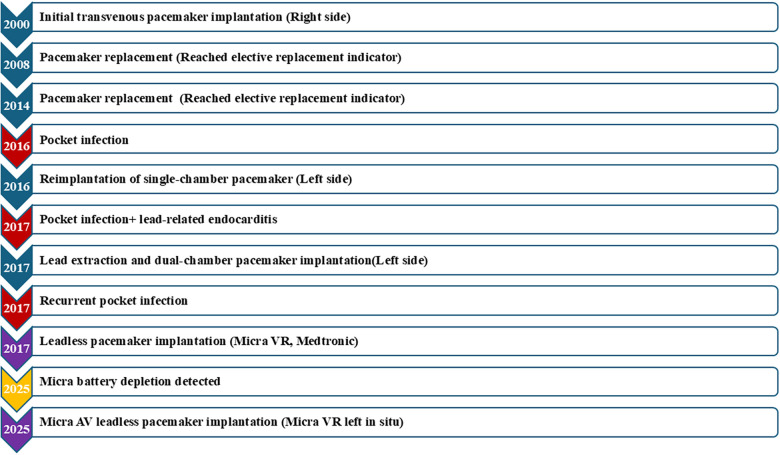
Clinical timeline of cardiac pacing history (2000–2026), including transvenous system implantations and revisions, recurrent device-related infections (2016–2017), transition to leadless pacing (Micra VR, 2017), and sequential leadless pacemaker implantation following battery depletion (Micra AV, 2025).

As the population of patients treated with leadless pacing increases, management of battery depletion has emerged as a new clinical challenge (6, 7). In individuals with a history of recurrent infection and prior transvenous lead extraction, repeated implantation of transvenous systems may be associated with substantial risk (3). In this context, a “leadless-only” strategy, involving implantation of a new device while leaving the depleted device *in situ*, may represent a feasible long-term approach (6). We present the application of a sequential leadless pacing strategy with transition from ventricular-only to atrioventricular-synchronous pacing using a Micra AV system (Figure 1). To our knowledge, this represents one of the first reported experiences in Central Asia.

## 2 Case description

A 65-year-old patient with a long-standing history of cardiac pacing spanning 26 years (2000–2026) presented in 2025 with battery depletion of a previously implanted leadless pacemaker (ventricular-only system, Micra VR, implanted in 2017). The clinical history was notable for recurrent device-related infections, including pocket infections and lead-related endocarditis, as well as multiple transvenous system revisions and prior transvenous lead extraction.

The patient's pacing history, clarified in detail in (Figure 1), comprised an initial transvenous pacemaker implantation in 2000 (right side), with generator replacements in 2008 and 2014 for elective replacement indicator. In 2016, a pocket infection occurred, requiring reimplantation of a single-chamber pacemaker on the left side. In 2017, the patient developed a further pocket infection with lead-related endocarditis, necessitating lead extraction and implantation of a dual-chamber pacemaker (left side); this was followed shortly thereafter by a recurrent pocket infection. Given this cluster of three infectious episodes within an 18-month period (2016–2017), each occurring in association with a transvenous system revision, the patient was transitioned to a leadless pacemaker (Micra VR) later in 2017. Notably, the leadless system remained free of infection throughout its 8-year service life; the exact causative organism or dominant patient-specific risk factor underlying the earlier transvenous-era infections was not definitively established, although the temporal clustering around repeated pocket manipulation and system revision is consistent with the recognized association between procedural frequency and cumulative cardiac implantable electronic device infection risk.

Battery depletion of the Micra VR was detected in 2025. Given the history of recurrent infections and prior transvenous system complications, reimplantation of a transvenous pacemaker was considered contraindicated. In addition, the patient had previously experienced significant difficulties related to transvenous pacing systems and declined further transvenous implantation. Extraction of the chronically implanted leadless device was not attempted. Chronically implanted leadless pacemakers are known to undergo endothelialization and fibrotic encapsulation, making late retrieval technically challenging and potentially associated with myocardial injury, with no clear clinical benefit in the absence of device-related infection (6, 8). Transthoracic echocardiography demonstrated no evidence of thrombus or vegetation related to the existing device.

The patient was pacemaker-dependent, with minimal or absent intrinsic ventricular rhythm, a consideration directly relevant to procedural planning as outlined in the manufacturer’s implant manual, Section 5.3.1 (9).

A decision was made to proceed with implantation of a Micra AV leadless pacemaker while leaving the previously implanted device *in situ* as part of a sequential leadless-only pacing strategy.

**Figure 2:**
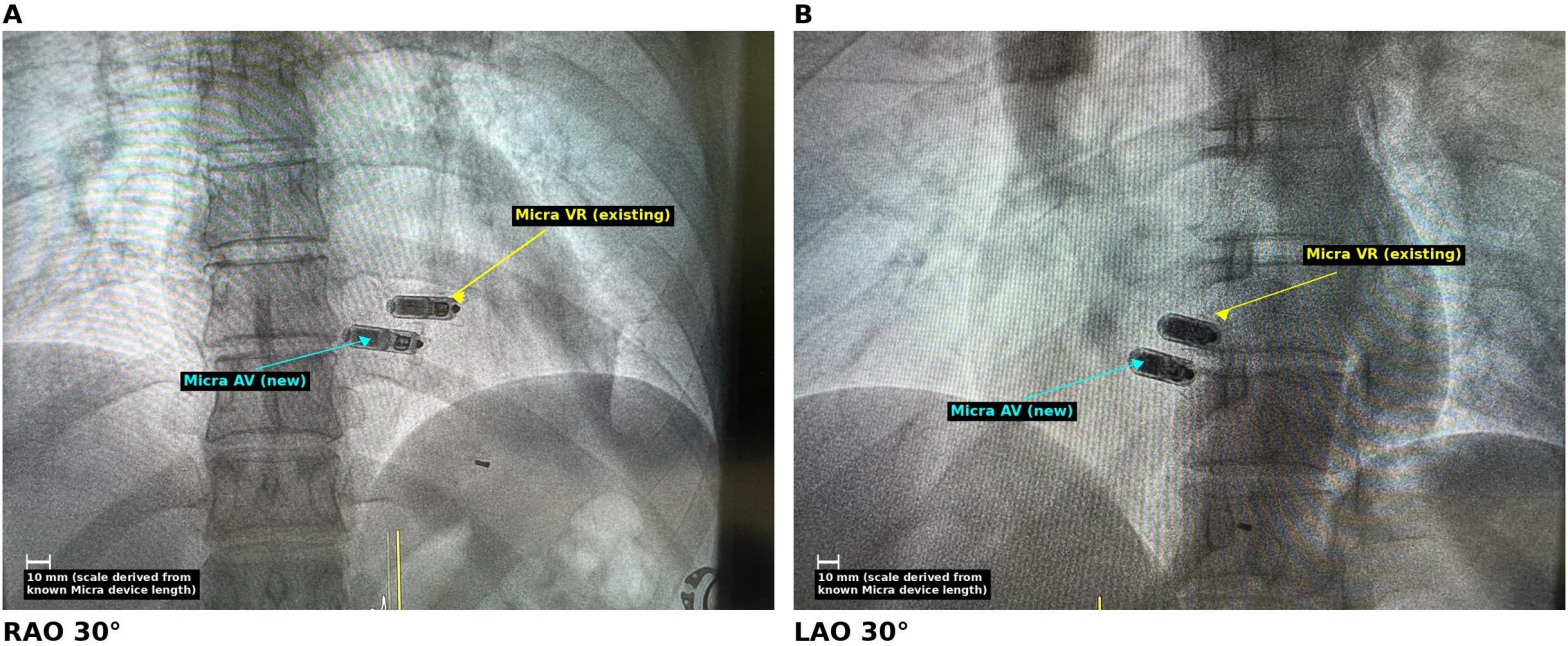
Final intraoperative fluoroscopic views confirming the position of both leadless devices and spatial separation between the chronically implanted Micra VR and the newly implanted Micra AV. **(A)** Right anterior oblique (RAO) 30° projection. **(B)** Left anterior oblique (LAO) 30° projection. Devices are labeled, and a scale bar (10 mm) derived from the known device length (25.9 mm) is provided in each panel.

To minimize infection risk in this high-risk patient, periprocedural antibiotic prophylaxis was administered. The procedure was performed under intravenous anesthesia via the right femoral vein with a 23-Fr delivery system, using a fully percutaneous, pocket-free approach. The delivery catheter was advanced into the right ventricle under fluoroscopic guidance, allowing visualization of the previously implanted depleted leadless pacemaker. The new device was implanted at the right ventricular mid-septum, selected under fluoroscopic guidance at a location visually distinct from the chronically implanted Micra VR, to ensure adequate spatial separation and minimize the risk of mechanical interaction (Figure 2); the chronically implanted device was left undisturbed throughout to avoid manipulation of previously affected tissue. Spatial separation and absence of mechanical interaction were assessed using multi-projection fluoroscopy (right and left anterior oblique views), without adjunctive echocardiographic confirmation, and inter-device distance was not formally quantified; this represents a limitation of our procedural approach.

Adequate fixation was confirmed using a standard pull-and-hold (“tug”) test. Acute electrical testing was performed to exclude device–device interference, particularly in relation to the accelerometer-based atrioventricular synchrony algorithm. Acute testing of the newly implanted Micra AV confirmed satisfactory pacing threshold (0.63 V), R-wave amplitude (9.8 mV), and impedance (720 *Ω*), with appropriate function of the accelerometer-based AV synchrony algorithm and no inappropriate sensing. Testing of the chronically implanted Micra VR at this procedure was limited to confirmation of its programmed backup parameters and fluoroscopic confirmation of its position; the pacing threshold, R-wave amplitude, and impedance of the Micra VR immediately prior to replacement (1.8 V, 6.5 mV, 560 *Ω*, respectively; Table 1) reflected its values at the time of battery depletion and were not formally retested at the second procedure. Intracardiac electrogram recording from the delivery catheter during the procedure showed no noise, far-field oversensing, or artifact attributable to the chronically implanted, electrically inactive Micra VR.

**Table 1:** Electrical parameters and atrioventricular synchrony of the leadless pacing system during follow-up.

Device (Model)	Time Point	Mode	Threshold (V@0.4 ms)	R-wave amplitude (mV)	Impedance (*Ω*)	AV Synchrony (%)
Initial Micra VR	Pre-replacement	VVI	1.8	6.5	560	Not applicable
Micra AV	Post-implant	VDD	0.63	9.8	720	80%
Micra AV	1-month	VDD	0.50	10.2	690	82%
Micra AV	3-month	VDD	0.50	9.7	670	84%
Micra AV	6-month	VDD	0.50	9.5	650	85%

Following successful implantation and confirmation of stable Micra AV function, the previously implanted Micra VR was programmed at the end of the procedure to a non-interfering backup mode (VVI, ventricular-paced, ventricular-sensed, inhibited, at 30 bpm) with minimal pacing output, rather than fully deactivated (“Device Off”) as specified in the manufacturer's implant manual, Section 5.3 (9). This deviation from the manufacturer's recommendation was a deliberate decision by the treating team: because the patient was pacemaker-dependent, a low-output ventricular backup was retained to provide a safety margin against asystole during the immediate post-implant period, until stable function of the newly implanted Micra AV could be confirmed, rather than leaving the patient with no ventricular pacing safety net. This trade-off — a small theoretical risk of competitive pacing or device–device interaction versus the risk of inadequate backup pacing in a pacemaker-dependent patient — is discussed further below.

**Figure 3:**
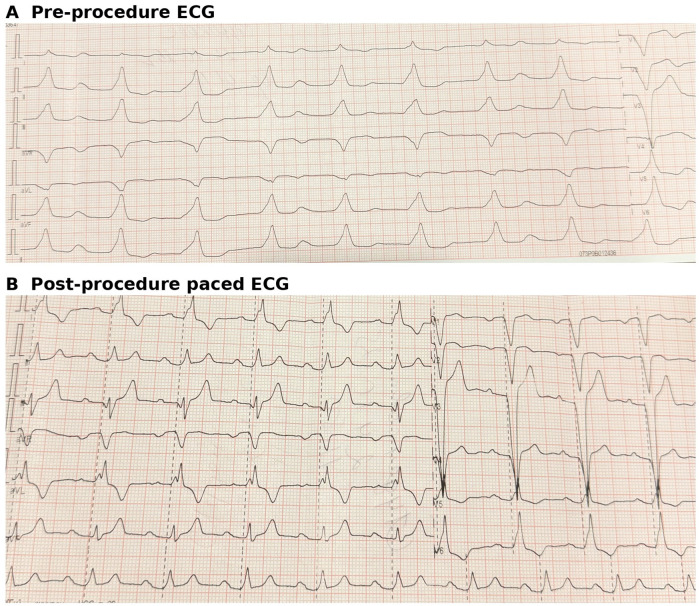
Twelve-lead surface ECG recordings before the procedure (A, baseline rhythm) and after Micra AV implantation during ventricular pacing **(B)** standard ECG recording parameters were a paper speed of 25 mm/s and a gain of 10 mm/mV. Lead labels (I, II, III, aVR, aVL, aVF, and V1–V6) are shown on the original tracings. Vertical dashed lines in panel B indicate ventricular pacing stimuli. No pacing artefacts were observed. We apologize for the image quality, as the ECG tracings were captured from printed paper records and higher-resolution scans were not available.

The 12-lead surface ECG was recorded before and after the procedure (Figure 3).

At follow-up, Micra AV performance remained stable. Detailed electrical parameters and atrioventricular synchrony are summarized in (Table 1). At 1 month, atrioventricular synchrony was 80%, with preserved rate responsiveness and stable sensing and impedance values. At 3 and 6 months, pacing thresholds remained at 0.50 V, impedance ranged from 670 to 650 *Ω*, and atrioventricular synchrony ranged from 84% to 85%. Assessment for device–device interaction at each visit consisted of interrogation of the Micra AV, including review of sensed/paced event counters, mode-switch episodes, and AV synchrony percentage, together with clinical assessment for symptoms suggestive of malfunction (palpitations, syncope, dizziness); no such evidence was identified. At each follow-up visit (1, 3, and 6 months), interrogation of the chronically implanted, backup-mode Micra VR confirmed stable electrical parameters (pacing threshold 0.5 V, impedance within normal range), in addition to interrogation of the newly implanted Micra AV, with no evidence suggestive of lead/circuit malfunction or device–device interaction. VVI pacing episodes of the retained Micra VR were not separately assessed (not applicable), as the device was programmed to a fixed VVI 30 bpm backup mode and served only as a low-output safety backup rather than an actively functioning system requiring serial reassessment of pacing burden. The patient was followed dynamically with clinical, device-interrogation, and biochemical assessment at each visit (comprising complete blood count and C-reactive protein measurement, with blood cultures obtained only if clinically indicated); no clinical or laboratory evidence of infection was identified at any time point.

No mechanical interaction or device-related complications, including infection or thrombus formation, were observed during the 6-month follow-up period. The proportion of ventricular pacing remained appropriate for the underlying pacing indication and stable throughout the observation period. The pacing threshold of the newly implanted device was lower compared to that of the previously implanted, depleted system.

## 3 Discussion

This case demonstrates the feasibility and safety of a sequential leadless-only pacing strategy in a patient with a history of recurrent device-related infection and prior transvenous system failure. The successful implantation of a second leadless pacemaker, while leaving the previously implanted device *in situ*, supports the use of leadless pacing in selected complex, high-risk clinical scenarios.

The management of patients with prior cardiac implantable electronic device infections remains challenging, particularly when repeated transvenous interventions are associated with a high risk of recurrent infection (3). Leadless pacemakers eliminate the need for a subcutaneous pocket and transvenous leads, thereby addressing the primary sources of infection (1, 2). Long-term registry data have also demonstrated sustained safety and performance of leadless pacemakers over extended follow-up periods (10). Although leadless pacing substantially reduces infection risk, it does not eliminate it entirely, and isolated cases of leadless pacemaker infection have been reported (11–13). In the present case, dynamic clinical, biochemical, and device-based follow-up over 6 months showed no evidence of infection, supporting the safety of this strategy even in a patient with a heavily infection-prone history. However, data on long-term management after battery depletion of leadless systems remain limited (6, 7).

In this context, a sequential leadless strategy may represent a reasonable approach. In the present case, adequate spatial separation and appropriate programming of the abandoned system were associated with stable device function without evidence of mechanical interaction or electrical interference. Importantly, despite the presence of a previously implanted leadless device within the right ventricle, atrioventricular synchrony based on the accelerometer-driven algorithm of the Micra AV was preserved, with reliable detection of atrial mechanical activity (A3/A4 signals). Atrioventricular synchrony was maintained at clinically relevant levels throughout follow-up.

The decision to avoid extraction of the chronically implanted Micra VR was based on the known risks of late retrieval, including the potential for myocardial injury due to fibrous encapsulation. In the absence of device-related infection, leaving the system *in situ* represents a reasonable strategy (6, 8). This case also illustrates the feasibility of upgrading from ventricular-only to atrioventricular-synchronous pacing using a leadless system (14).

Although extraction of tine-fixation leadless pacemakers such as the Micra is technically more feasible than retrieval of fixed-helix systems, and has been reported even after multi-year dwell times using snare-based techniques (15, 16), late extraction nonetheless carries non-negligible risks, including tricuspid valve injury, myocardial perforation, and vascular trauma related to traction on a chronically endothelialized device. The leave-in-situ strategy avoids these procedural risks but carries the theoretical long-term risks of multiple intracardiac devices, including increased thrombogenic surface area and progressive occupation of right ventricular space. In the present case, in the absence of infection involving the chronically implanted device, and given the patient's wish to avoid any further invasive procedure, leave-in-situ was judged to offer a more favorable risk–benefit balance than elective extraction. We acknowledge that extraction remains a reasonable alternative in tine-fixation systems and that this decision should be individualized based on dwell time and operator experience with retrieval techniques.

For this patient, transition to a retrievable helix-fixation system (Aveir, Abbott) was considered but not pursued: achieving AV synchrony with this platform would have required an additional atrial leadless device, further increasing the burden of chronically implanted hardware in a patient with a complex infection history, and the patient categorically declined transition away from the Micra platform with which the patient had remained complication-free for 8 years. Nonetheless, for younger patients with substantial life expectancy who may eventually require a third leadless device, retrievable helix-fixation systems warrant consideration at the time of the second implantation, as they preserve the option of extraction and may help mitigate progressive intracardiac device accumulation.

Based on the spatial separation achieved in this case and typical right ventricular dimensions, it is plausible that one, and in select anatomically favorable patients possibly two, additional leadless devices could be accommodated using a leave-in-situ strategy before right ventricular space, vascular access, or device–device interaction become limiting. However, this remains speculative in the absence of dedicated anatomical or modeling studies, and we suggest this as a priority area for future research rather than a number that can be reliably predicted from a single case.

Epicardial pacing represents an alternative option in patients with recurrent cardiac implantable electronic device infections (17). However, in this case, it was considered less favorable due to the need for a more invasive surgical approach and the creation of an additional generator pocket, which may increase the risk of infection.

Looking ahead, the long-term management of patients undergoing sequential leadless pacing remains an evolving clinical consideration (6, 7). The feasibility of additional device implantation will depend on factors such as spatial constraints within the right ventricle, device–device interaction, and overall clinical context. Further studies are needed to better define optimal long-term strategies in such patients.

In summary, this longitudinal clinical experience suggests that a sequential leadless-only pacing strategy may represent a feasible and safe long-term approach in patients with a high risk of recurrent device-related infections. Our experience with leadless pacing over 8 years supports the feasibility of a “leave-in-situ” approach for depleted systems, avoiding the potential risks associated with device extraction (6). This sequential strategy allows therapeutic continuity and enables transition to atrioventricular-synchronous pacing, providing a clinically relevant option in complex patients.

### 3.1 Limitations

This report has several limitations. First, confirmation of spatial separation between the two devices relied on fluoroscopic visualization in two projections (RAO and LAO); adjunctive echocardiography and formal quantification of inter-device distance were not performed. Future procedures of this kind may benefit from echocardiographic confirmation and formal measurement of inter-device distance. Second, the exact causative organism or dominant patient-specific risk factor underlying the earlier transvenous-era infections could not be definitively established. Third, follow-up assessment for device–device interaction relied primarily on interrogation of the newly implanted Micra AV (event counters, mode-switch episodes, AV synchrony percentage); although the retained Micra VR was also interrogated at each visit, confirming stable pacing threshold and impedance, VVI pacing episodes of this backup-mode device were not separately quantified, as it was not considered an actively functioning pacing system requiring serial reassessment of pacing burden. Fourth, the chronically implanted Micra VR was left in a low-output backup (VVI 30) rather than fully deactivated mode, a deliberate deviation from the manufacturer's implant manual recommendation in this pacemaker-dependent patient, as discussed above; this trade-off should be considered individually in similar cases. Fifth, as a single-patient case report, the generalizability of this sequential leadless-only strategy requires confirmation in larger series with longer follow-up.

## 4 Patient perspective

The patient reported satisfaction with the leadless pacing strategy and improvement in quality of life after the procedure. The patient had previously experienced significant difficulties related to transvenous pacing systems and therefore declined further transvenous implantation. Avoidance of additional transvenous leads and surgical pocket formation was considered particularly important given the history of recurrent infections and multiple procedures.

## Data Availability

The original contributions presented in the study are included in the article/Supplementary Material, further inquiries can be directed to the corresponding author/s.
